# Optimizing macrolide resistance detection for *Mycobacterium abscessus*: a potential low-cost, time-saving alternative

**DOI:** 10.1128/spectrum.00942-25

**Published:** 2025-09-08

**Authors:** Liang-En Hwang, Aristine Cheng, Hsin-Yun Sun, Jung-Yien Chien, Po-Ren Hsueh, Wang-Huei Sheng

**Affiliations:** 1 Department of Medicine, National Taiwan University Hospital and College of Medicine 38006, Taipei, Taiwan; 2 College of Medicine, China Medical University 568605, Taichung, Taiwan; Taichung Veterans General Hospital, Taichung, Taiwan, Province of China

**Keywords:** *Mycobacterium abscessus*, microbial sensitivity tests, macrolides, anti-bacterial agents, Taiwan

## Abstract

*Mycobacterium abscessus* complex (MABC) is notoriously difficult to treat. Current guidelines suggest a 14-day-long incubation and/or sequencing of *erm41* to detect inducible macrolide resistance. We assessed whether the evolution of minimum inhibitory concentrations (MICs) can reliably predict inducible macrolide resistance and clinical outcomes of extrapulmonary MABC infections. Between 2013 and 2015, *M. abscessus* isolates were identified from extrapulmonary sites at a medical center. Drug susceptibility testing (DST) was conducted with results read on D3, D7, and D14. A fourfold or more increase in clarithromycin MIC between D3 and D7 was used to predict inducible macrolide resistance. The results were compared with the genotypic prediction of DST using sequencing of *erm41* and *rrl*. Clinical data were reviewed. Thirty-five unique *M. abscessus* isolates comprising 16 subsp. *abscessus*, 18 subsp. *massiliense,* and 1 subsp. *bolletii* were identified. The overall inducible macrolide resistance was 40%. Using the proposed prediction rule, the sensitivity and specificity were 81% and 100%, while using genotypic prediction, the sensitivity and specificity were 75% and 100%, respectively. From the chart review, 28 patients received treatment, among whom 18 had evaluable outcomes. Most (92.9%) of the patients received macrolide-based combinatorial treatment; 71.4% of them received surgery. Of the outcome-evaluable patients, 77.8% had favorable outcomes. Our preliminary study highlighted that susceptibility testing read within one week holds promising potential for detecting inducible macrolide resistance. Further validation with larger cohorts of *M. abscessus* pulmonary and extrapulmonary disease is warranted.

**IMPORTANCE:**

Drug susceptibility testing for difficult-to-treat microorganisms like *Mycobacterium abscessus* is frequently unavailable due to costs and technical demands. We propose a potential low-cost, time-saving alternative, using interval minimum inhibitory concentration evolution to predict the final phenotypic results. Our preliminary findings suggest comparable performance of the proposed early prediction method to the gold standard and the genotypic prediction in our small, extrapulmonary *M. abscessus* complex collections. Given its promising potential, validation in a larger cohort with pulmonary disease is needed for wider clinical application.

## INTRODUCTION

Nontuberculous mycobacteria (NTM) are ubiquitous in the environment and are recognized as human pathogens (1). The disease burden mainly lies in pulmonary disease in those with pre-existing lung pathology; however, some less common manifestations, such as skin and soft tissue infections (SSTIs), ocular and otic infections, or disseminated infections in adult-onset immunodeficiency syndromes are observed (2). The incidence of NTM infections is on the rise, due to improvement in awareness, diagnostics, and expanding population of immunocompromised hosts (1, 3).

Notoriously regarded as one of the most resistant pathogens, *Mycobacterium abscessus* complex (MABC) is phylogenetically classified into three distinct subspecies, *M. abscessus* subspecies *abscessus*, *M. abscessus* subspecies *massiliense,* and *M. abscessus* subspecies *bolletii* (referred to as *M. abscessus, M. massiliense,* and *M. bolletii* hereafter.) *M. massiliense* is characterized by its truncated erythromycin ribosome methyltransferase gene (*erm41*) from a frame-shift mutation, rendering it more susceptible to clarithromycin (4). The other two subspecies possess inducible macrolide resistance courtesy of a functional *erm41* that can negatively impact treatment outcomes in pulmonary disease (5). Initially described by Nash et al., *erm41* C28 sequevar (T-to-C transition at position 28) reverses inducible macrolide resistance (6). While adenine point mutations in a region of the *rrl* gene that encodes a peptidyltransferase domain of the 23S rRNA at either position 2058 (A →G, C, or T substitution) or 2059 (A →G, C, or T substitution) confer acquired macrolide resistance (7).

Currently, the Clinical and Laboratory Standards Institute (CLSI) still recommends clinical laboratories to report results of drug susceptibility test (DST) after 14 days of incubation using standard broth dilution methods (8). The practice is time-consuming, leading some to suggest routine sequencing of *erm41* and *rrl* on MABC to facilitate diagnosis (9). However, genotypic resistance correlates imperfectly with phenotypic resistance (10). Christianson et al. tested 104 MABC isolates and reported that the majority (82.7%) of the macrolide-resistant isolates were already resistant by day 7, thus questioning the added value of such a lengthy incubation, but their findings are yet to be replicated (11). An *in vitro* study by Choi et al. also showed that MABC isolates demonstrated high levels of *erm41* mRNA expression with corresponding minimum inhibitory concentration (MIC) changes by day 7, with the maximal mRNA expression peaking at day 3 when testing with clarithromycin and peaking at day 7 when testing with azithromycin (5). Similarly, the acquisition of an *in vitro* resistant phenotype by *Escherichia coli* isolates due to inducible chromosomal AmpC beta-lactamase also appears to be evident by day 3, unlike *ampC* knockout mutants or mutants whose *ampC* had been moved to a different location in the chromosome. Although AmpC activity and MICs for amoxicillin continue to increase until day 19 for wild-type isolates with *ampC* that can be transcriptionally activated, the MICs had diverged by day 3 (12). Thus, using MIC evolution to predict inducible resistance mechanisms that cumulate into a maximal MIC peak later may not be an unreasonable time-saving approach.

Regarding treatment, current guidelines support macrolide-based combination therapy for MABC pulmonary infection irrespective of macrolide susceptibility, as it offers immunomodulatory effect even when a macrolide-resistant strain is implicated (13). However, due to the rarity of extrapulmonary infections, the association between macrolide susceptibility and treatment outcomes is more elusive (14, 15).

There are two aims in the study; first, we postulated that the fold changes in macrolide MICs between day 3 and day 7 may serve as a predictor for inducible macrolide resistance. Second, since extrapulmonary MABC cohorts are less often studied compared to pulmonary MABC cohorts, we aimed to describe the outcomes of such a cohort in relation to host clinical and microbiological characteristics.

## RESULTS

**Fig 1:**
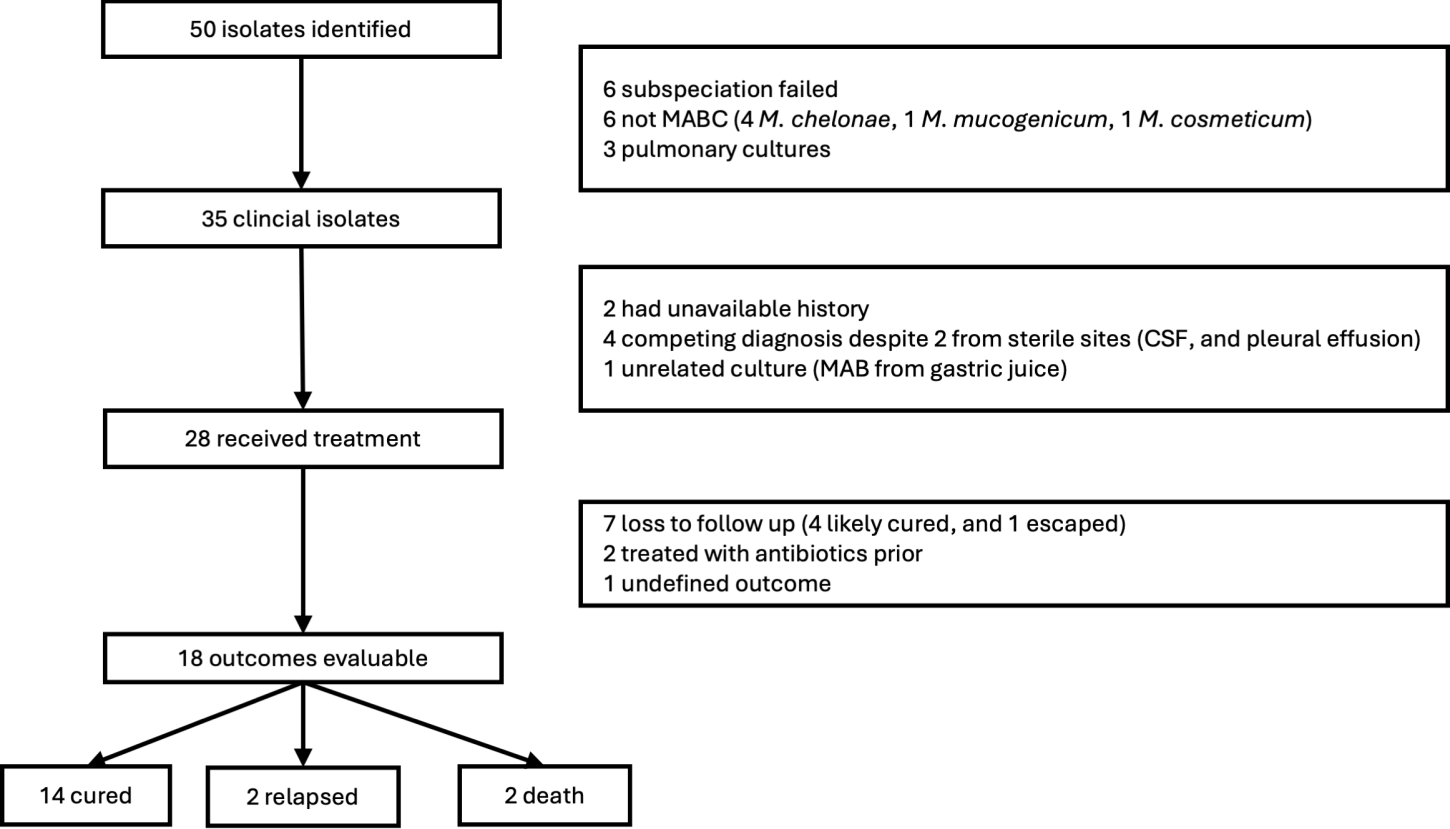
The overall cohort and outcome-evaluable cohort.

Between 2013 and 2015, 50 extrapulmonary MABCs were identified from routine clinical laboratory (Fig. 1). Among these, six isolates could not be assigned unambiguously to a subspecies, another six were subsequently identified as non-MABC, and another three were found to be pulmonary-related specimens. Ultimately, 35 unique *M. abscessus* isolates from extrapulmonary sites were eventually analyzed (Fig. 1), comprising 16 (45.7%) *M*. *abscessus*, 18 (51.4%) *M*. *massiliense*, and 1 (2.9%) *M*. *bolletii*.

### Antibiotic susceptibility testing

The drug susceptibility results using broth microdilution are shown in Table 1. *M. massiliense* demonstrated fair *in vitro* susceptibility to clarithromycin, with 14 (77.8%) of the isolates remaining susceptible at day 14. Amikacin susceptibility was equally high among *M. abscessus* isolates and *M. massiliense* (81.3% vs 88.9%), with only one isolate classified as resistant. The isolates were variably susceptible to cefoxitin, imipenem, doxycycline, minocycline, and linezolid, as shown. No isolates were resistant to tigecycline, and none were susceptible to quinolones.

**TABLE 1:** MICs of the included *Mycobacterium abscessus* isolates against selected antibiotics and breakpoints interpretation (*N* = 35)^*a*^^,^^*b*^

Isolate and antibiotic	MIC 50	MIC 90	MIC range	S	I	R
*M. abscessus* subsp. *abscessus* (mg/L, *N* = 16)						
Clarithromycin D3	0.5	8	0.12–16	14 (87.5)	0 (0)	2 (12.5)
Clarithromycin D7	16	32	0.5–32 (>16)	6 (37.5)	1 (6.3)	9 (56.3)
Clarithromycin D14	32	32	1–32 (>16)	2 (12.5)	1 (6.3)	13 (81.3)
Amikacin	8	32	8–128 (>64)	13 (81.3)	2 (12.5)	1 (6.3)
Cefoxitin	32	128	32–128	0 (0)	15 (93.8)	1 (6.3)
Imipenem	16	32	4–32	1 (6.3)	14 (87.5)	1 (6.3)
Ciprofloxacin	8	8	2–8 (>4)	0 (0)	2 (12.5)	14 (87.5)
Moxifloxacin	16	16	4–16 (>8)	0 (0)	0 (0)	16 (100)
Doxycycline	32	32	4–32 (>16)	0 (0)	1 (6.3)	15 (93.8)
Minocycline	16	16	8–16 (>8)	0 (0)	0 (0)	16 (100)
Tigecycline	1	1	0.06–1	6 (37.5)	10 (62.5)	0 (0)
Linezolid	32	32	4–64 (>32)	2 (12.5)	4 (25.0)	10 (62.5)
TMP/SMX	>8/152	>8/152	>8/152	0 (0)	0 (0)	16 (100)
*M. abscessus* subsp. *massiliense* (mg/L, *N* = 18)						
Clarithromycin D3	0.25	0.5	0.12–1	18 (100)	0 (0)	0 (0)
Clarithromycin D7	0.5	1	0.12–32 (>16)	17 (94.4)	0 (0)	1 (5.6)
Clarithromycin D14	1	8	0.12–32 (>16)	14 (77.8)	2 (11.1)	2 (11.1)
Amikacin	16	32	4–32	16 (88.9)	2 (11.1)	0 (0)
Cefoxitin	32	128	32–128	0 (0)	16 (88.9)	2 (11.1)
Imipenem	16	32	8–64	0 (0)	14 (77.8)	4 (22.2)
Ciprofloxacin	8	8	4–8 (>4)	0 (0)	0 (0)	18 (100)
Moxifloxacin	16	16	2–16 (>8)	0 (0)	1 (5.6)	17 (94.4)
Doxycycline	32	32	16–32 (>16)	0 (0)	0 (0)	18 (100)
Minocycline	16	16	2–16 (>8)	0 (0)	2 (11.1)	16 (88.9)
Tigecycline	1	1	0.25–1	7 (38.9)	11 (61.1)	0 (0)
Linezolid	32	32	2–64 (>32)	4 (22.2)	2 (11.1)	12 (66.7)
TMP/SMX	>8/152	>8/152	>8/152	0 (0)	0 (0)	18 (100)

^*a*^ The only *M. abscessus *subsp.* bolletii* had clarithromycin MIC of 2 mg/L (S), 32 mg/L (R), 32 mg/L (R) on D3, D7, and D14, and amikacin MIC of 8 mg/L (S), cefoxitin 32 mg/L (S), imipenem 8 mg/L (I), moxifloxacin 8 mg/L (R), ciprofloxacin 8 mg/L (R), doxycycline 32 mg/L (R), tigecycline 0.5 mg/L (S), linezolid 8 mg/L (S), and TMP/SMX > 8/152 mg/L (R).

^*b*^ For clarithromycin, the respective breakpoints categorized as susceptible, intermediate, and resistant were MIC ≤2, 4, and ≥8 mg/L; for amikacin, the breakpoints were ≤16, 32, and ≥64 mg/L; for cefoxitin, 16, 32–64, and ≥128 mg/L; for imipenem ≤4, 8–16, and ≥32 mg/L; for moxifloxacin, ≤1, 2, and ≥4 mg/L; for ciprofloxacin, ≤1, 2, and ≥4 mg/L; for doxycycline, ≤1, 2–4, and ≥8 mg/L; for linezolid, ≤8, 16, and ≥32 mg/L, to be categorized as susceptible, intermediate, and resistant. For TMP/SMX, ≤2/38 mg/L was categorized as susceptible, and ≥4/76 mg/L was categorized as resistant; these breakpoints were interpreted per the CLSI guidance. For minocycline, the breakpoints were ≤1, 2–4, and ≥8 mg/L, borrowed from standards for slowly growing mycobacterium, and tigecycline they were ≤0.5, 1, and ≥2mg/L, per previous studies.

### Evolution of MIC and prediction of inducible macrolide resistance

The correlations between early predicted inducible macrolide resistance, genotypic resistance, and inducible resistance by the standard broth microdilution method are shown in Table 2. Of all 35 isolates, 16 (45.7%) were phenotypically resistant to clarithromycin at day 14, including 2 with baseline resistance (resistant on D3). However, only 11 (68.6%) were phenotypically resistant to clarithromycin at day 7, and only 2 (12.5%) were resistant at day 3. Using the proposed prediction method, 18 of all isolates had ≥4-fold increase in MIC between D3 and D7, among which 12 isolates with “early predicted inducible macrolide resistance” had an MIC ≥2 mg/L by day 7, correctly classifying 12 out of 14 isolates with inducible macrolide resistance (85.7%). The overall sensitivity was 81.3% in identifying resistant isolates, and the specificity was 100% in identifying susceptible isolates. The results are comparable with genotypic prediction among our small cohort, showing 75.0% sensitivity and 100.0% specificity and superior to day 7 MIC alone.

**TABLE 2:** Comparisons of proposed “early predicted inducible macrolide resistance” based on MIC evolution, genotypic prediction, and phenotypic drug susceptibility testing results (*N* = 35)

Parameter	Phenotypic D14 macrolide resistance	Phenotypic D3 macrolide resistance	Phenotypic D7 macrolide resistance	Early predicted macrolide resistance	Genotypic macrolide resistance
Classified as resistant	16	2^*a*^	11	12 + 1^*b*^	12
Classified as non-resistant	19	33	24	22^*b*^	23
Inducible resistance	14	0	9	12	12^*c*^
Very major error^*d*^		14	5	3	4
Major error^*d*^		0	0	0	0
Sensitivity^*e*^		12.5% (2/16)	68.8% (11/16)	81.3% (13/16)	75.0% (12/16)
Specificity^*f*^		100.0% (33/19)	100.0% (24/19)	100.0% (22/19)	100.0% (23/19)

^*a*^ Includes 2 isolates with acquired resistance (phenotypically resistant since D3) and 33 non-resistant isolates on D3.

^*b*^ Eighteen isolates had a fourfold increase in MIC, including 1 with baseline resistance (MIC from 8 to 32 mg/L), 12 with inducible resistance, 5 eventually non-resistant. The other isolate with baseline resistance had MIC change from 16 to 32 mg/L.

^*c*^ No isolate is predicted to have baseline resistance since no mutations in *rrl *A2058/2059 were present.

^*d*^ Very major error was defined as predicted susceptible but eventually resistant on D14 DST, whereas major error was defined as predicted resistant yet with MIC <8 mg/L on D14.

^*e*^ Sensitivity was the percentage of those (predicted) resistant among truly resistant isolates on D14.

^*f*^ Specificty was the percentage of those (predicted) non-resistant among the truly non-resistant isolates on D14.

The proposed prediction method resulted in three very major errors (incorrectly predicted susceptible but ultimately resistant) and no major error (predicted resistant but ultimately sensitive). The detailed bacteriologic characteristics were shown in Table 3. The same three isolates also fell into the very major error category using the genotypic method and the day 7 MIC alone. An additional *M. massiliense* was misclassified by the genotypic method as susceptible, and two additional *M. abscessus* were classified as non-resistant by the day 7 MIC alone (Table 3).

**TABLE 3:** Microbiology characteristics of the isolates with very major error from the early predicted method, genotypic method, and isolated ≥4-fold increase in MIC

Isolate ID	Subspecies	D3 clarithromycin MIC (mg/L)	D7 clarithromycin MIC (mg/L)	D14 clarithromycin MIC (mg/L)	Erm41 sequevar
Very major error by early predicted macrolide resistance (*N* = 3)					
7	*M. abscessus*	0.12	1	16	C28
11	*M. abscessus*	0.5	1	16	C28
26	*M. massilliense*	0.5	1	16	Truncated
Very major error by genotypic method (*N* = 4)					
7	*M. abscessus*	0.12	1	16	C28
11	*M. abscessus*	0.5	1	16	C28
26	*M. massilliense*	0.5	1	16	Truncated
34	*M. massilliense*	2	32	32	Truncated
Very major error by D7 MIC alone (*N* = 5)					
1	*M. abscessus*	0.5	2	16	T28
7	*M. abscessus*	0.12	1	16	C28
10	*M. abscessus*	0.25	4	32	T28
11	*M. abscessus*	0.5	1	16	C28
26	*M. massilliense*	0.5	1	16	Truncated
Isolates with a ≥4-fold MIC evolution from D3 to D7, but with D7 clarithromycin MIC ≤1 mg/L (*N* = 5)					
14	*M. abscessus*	0.25	1	1	C28
22	*M. massilliense*	0.12	1	1	Truncated
31	*M. massilliense*	0.12	0.5	0.5	Truncated
32	*M. massilliense*	0.12	1	2	Truncated
37	*M. massilliense*	0.06	0.25	0.5	Truncated

### Description of the cohort

Of the 35 patients, 28 (80.0%) received treatment (Fig. 1). For the seven patients who did not receive antimicrobial treatment, the majority had competing diagnoses leading to uncertainties as to whether the MABC was a true pathogen or resolution of symptoms and signs after curative surgery.

 The demographic and clinical characteristics of treated and untreated patients are summarized in Table 4. The median age of the treated population was 45 years, and 17 (60.7%) were women. The pathogenic subspecies comprised 15 (53.6%) *M*. *abscessus*, 12 (42.9%) *M*. *massilliense*, and 1 (3.6%) *M*. *bolletii*. For the seven untreated isolates, six of them were *M. massiliense*. The predominant sites of infection in decreasing order of frequency were skin and soft tissue (*n* = 10, 35.7%), otic (*n* = 7, 25.0%), bone (4, 14.3%), and eye (2, 7.1%).

**TABLE 4:** Patient characteristics of those who received treatment for *Mycobacterium abscessus* extrapulmonary infections

Characteristic	All patients (*n* = 35)	Patients who received treatment (*n* = 28)
Age, years (median, interquartile range, IQR)	46 (33–58)	45 (33.5–54)
Sex		
Male	14 (40.0)	11 (39.3)
Female	21 (60.0)	17 (60.7)
Presence of anti-interferon γ autoantibody	2 (5.7)	2 (7.1)
History of cancer	4 (11.4)	3 (10.7)
Receipt of solid organ transplant	3 (8.6)	3 (10.7)
*M. abscessus* subspecies		
subsp. *abscessus^a^*	16 (45.7)	15 (53.6)
subsp. *massiliense*	18 (51.4)	12 (42.9)
subsp. *bolletei*	1 (2.9)	1 (3.6)
Infected sites		
Lymphadenitis	3 (8.6)	3 (10.7)
Otic	8 (22.9)	7 (25.0)
Skin and soft tissue infection	11 (31.4)	10 (35.7)
Bone	4 (11.4)	4 (14.3)
Ocular	2 (5.7)	2 (7.1)
CNS	2 (5.7)	1 (3.6)
Others^*b*^	5 (14.3)	1 (3.6)
Prosthesis involved	6 (17.1)	6 (21.3)
Treatment		
Surgery/debridement involved		20 (71.4)
<2 effective treatments		11 (39.3)
Two or more effective treatments		15 (53.6)
Topical treatment only		2 (7.1)
Regimen		
Macrolides		26 (92.9)
Any quinolone^*c*^		20 (71.4)
Amikacin		15 (53.6)
Doxycycline		11 (39.3)
Imipenem		9 (32.1)
Tigecycline		8 (28.6)
Linezolid		8 (28.6)
TMP/SFX		5 (17.9)
Treatment duration, months		7 (4.5–12.5)
Outcome		
Evaluable		18
Cured		14 (50.0)
Death before documentation of cure		2 (7.1)
Relapsed		2 (7.1)
Non-evaluable		10
Lost to follow-up		7 (25.0)
Receiving NTM treatment prior to diagnosis		2 (7.1)
Undefined outcome		1 (3.6)

^*a*^ There were 11 sequevar T28 and 5 C28* M. abscessus *isolates, among which 11 T28 and 4 C28 sequavars received treatment.

^*b*^ One subject with end stage kidney disease (ESKD) under peritoneal dialysis had isolated culture within ascites, one man had MAB growth in pleural effusion, and one patient had liver abscess and one patient had positive culture in gastric juice.

^*c*^ Fourteen (50%) patients got moxifloxacin, 13 (46.4%) patients got levofloxacin and 5 (17.9%) got ciprofloxacin.

Regarding treatment, 20 (71.4%) had undergone surgery for infection control, 26 (92.9%) were prescribed macrolide-based treatment, and 15 (53.6%) received amikacin. For outcomes, 18 (64.3%) of them had evaluable outcomes, including 14 (50.0%) who were cured without relapse. For the four (14.3%) patients with unfavorable outcomes, two of them died before documentation of cure, including one with neutralizing anti-interferon-γ autoantibodies (AIGAs), and the other had *M. abscessus* sternal wound infection after heart transplantation. The other two patients had relapses, including one with neutralizing AIGA, while the other had difficult-to-treat otomastoiditis.

### Comparisons of patients with favorable and unfavorable outcomes

There were more patients aged over 50 years in the unfavorable outcome group (28.6% vs 75.0%) (Table S1). Both patients with AIGA had unfavorable outcomes; one died before documentation of cure, and the other relapsed. Patients with *M. massiliense* infections had better outcomes than *M. abscessus* infections (57.1% vs 25.0%), but due to the small numbers of patients in each group, the difference was not statistically significant (*P* = 0.45). Regarding infection sites, we found that only two patients with SSTIs had evaluable outcomes, and both patients with ocular infections had favorable outcomes. Five of the seven treated otic infections had favorable outcomes. Surgical debridement appeared slightly more frequent among those with favorable outcomes (85.7% vs 50.0%, *P* = 0.20), but clarithromycin susceptibility did not trend with favorable outcomes (57.1% vs 75.0%, *P* = 1.0).

## DISCUSSION

Extrapulmonary MABC infections are uncommon (16, 17). Susceptibility testing is recommended, but not uniformly performed. For example, Kumar et al. reviewed 39 case reports/series on NTM extrapulmonary infections and only 26 of them had DST reported (18). The method itself is problematic, and inherent technical difficulties abound, such as endpoint reading and trailing, drug stability for extended incubation, availability of local expertise, and absence of established breakpoints (9). We used the evolution of clarithromycin MIC between day 3 and day 7 to predict inducible macrolide resistance and correlated it with the final day 14 phenotypic results. The prediction rule has a theoretical basis, as Choi et al. challenged reference *M. abscessus* strain to different levels of clarithromycin and found that *erm41* mRNA expression followed a dose-dependent manner to environmental macrolides concentration and peaked on day 3, and 75% of the isolates had demonstrated macrolide resistance by day 7 (5). The proposed new method, performed on our small cohort, was 81.3% sensitive for true macrolide resistance and 100.0% specific for macrolide susceptibility when predicted resistance was defined as ≥4-fold evolution in MIC with the absolute D7 MIC value ≥2 mg/L. The sensitivity would increase to 87.5% if only the ≥4-fold MIC evolution rule was applied, with decreasing specificity at 73.7% (Table 3). Caution is warranted in applying our findings, as the observation is based on a relatively small, extrapulmonary cohort, and a single misclassification could decrease sensitivity from 81.3% to 75.0%.

For reducing turnaround times to facilitate more rapid antimicrobial tailoring, the European Committee on Antimicrobial Susceptibility Testing has developed a rapid disc diffusion method using direct suspension from blood culture bottles with interpretation of inhibition zone diameters performed within 4, 6, and 8 h of incubation. The proportion of isolates with major error and very major error to standard approach decreased with longer incubation times (19). Traditional DST to macrolide concludes by day 14 if the isolates remain susceptible. Christianson’s study suggested that the addition of a day 7 read could greatly reduce the turnaround time, with minor effect on missing those with inducible resistance, as 87.2% of inducible macrolide resistance was already evident in the 104 MABC isolates they collected (11). The lower predictive value at 68.8% using only a day 7 read in our study was enhanced by using the evolutionary MIC prediction method, allowing identification of an additional two isolates in our cohort.

We sequenced *erm41* and *rrl* genes to predict phenotypic resistance, as increasing evidence suggests genotypic testing in *rrl* alongside *erm41* sequevar could correlate well with final DST results (9, 20, 21). Unfortunately, no *rrl* A2058/A2059 mutations were evident. The *M. abscessus* isolates in the very major error group by both our early prediction method and the genotypic method belonged to the C28 sequevar (Table 3). This is perhaps unsurprising as other studies have observed a wide range of MIC distribution within the same *erm41* sequevars without *rrl* mutations, suggesting the possibility of heteroresistant populations or complicated underlying regulatory determinants of *erm41* (22–24). On the other hand, *M. massiliense* isolates may acquire inducible macrolide resistance through horizontal gene transfer (25).

We found that *M. massiliense,* as opposed to *M. abscessus* infections, was disproportionately left untreated, suggesting that these isolates were potentially regarded as healthcare-associated contaminants or colonizers (26, 27). Overall, 14 (50%) patients achieved “cure,” but since 6 patients with SSTIs were classified as lost to follow-up because they neither returned after treatment completion nor had evident relapse, the actual cure rate is likely higher. The only variable that significantly affected treatment outcome was the presence of neutralizing AIGA. In previous cohorts studying patients with neutralizing AIGA and NTM infections, only 9 out of 45 (20%) patients received temporary cure, although the definition was different from ours (28). Screening for immunodeficiency and dissemination should be considered in patients with MABC SSTI (29). A standard endpoint is necessary to compare treatment outcomes across studies.

There are considerable limitations to our small, single-center study. The preliminary report of sensitivity and specificity using the proposed early prediction method needs to be validated in larger, preferably multi-center collections of MABC before clinical applications. The analysis was also performed solely on extrapulmonary isolates, which further limits the generalizability. For the clinical part, our cohort was heterogeneous, with variable host immunity, sites of infections, severity, and management strategies. Certain treatments may be more appropriate in specific scenarios, for example, surgery for otic infections rather than disseminated infections. Many patients with uncomplicated skin and soft-tissue infections could not be evaluated due to loss to follow-up, possibly indicating cure post-treatment. Although challenging, larger studies with more uniform disease groups are needed to inform better practice.

In conclusion, our proposed prediction method may be a promising tool for detecting inducible macrolide resistance with the obvious advantages of shortening turnaround time for DST, and decreasing costs and accessibility of genotyping, allowing timely regimen adjustments and precision care. However, application in routine practice warrants caution until validated in larger cohorts, particularly those including pulmonary isolates, representing greater disease burden from MABC. From our cohort, treatment outcomes depended more on underlying host immunity, surgery, and less on antimicrobial choice. Further research on translating MIC changes to guide MABC therapy for pulmonary MABC disease is warranted.

## MATERIALS AND METHODS

### Background and laboratory methods

Non-duplicate MABC isolates were collected from patients for routine clinical laboratory processing between 2013 and 2015 at the National Taiwan University Hospital, Taipei, Taiwan. Isolates from pulmonary origin were excluded. In 2013, *M. abscess* complex was distinguished through multilocus gene sequencing of three housekeeping genes, *hsp65*, *rpoB*, and *secA*, as described previously (30). Since 2014, our clinical laboratory has employed matrix-assisted laser desorption ionization-time of flight mass spectrometry (MALDI-TOF, MALDI Biotyper system, Microflex LT, Bruker Daltonik, GmbH, Bremen, Germany) routinely for speciation, and information on subspecies was provided simultaneously. Sequencing of erm41 and *rrl* gene was done as previously described by Lee et al. (31).

### Drug susceptibility testing (DST) and prediction of inducible macrolide resistance

Broth microdilution was performed with isolates incubated in cation-adjusted Mueller-Hinton broth, with breakpoints interpreted according to the CLSI standards (32). Minocycline breakpoints were interpreted for slowly growing mycobacteria, while the tigecycline breakpoint was adopted from a previous study (33). The MIC50 and MIC90 were defined as the drug level that inhibited 50% and 90% of the isolates of the same subspecies. For clarithromycin, we performed MIC reading on days 3, 7, and 14. Inducible macrolide resistance was defined by those with clarithromycin MIC <8 mg/L on day 3, but turned resistant (MIC ≥ 8 mg/L) by day 14. Acquired macrolide resistance was defined as isolates that appeared resistant to clarithromycin from day 3 onwards.

We proposed a new prediction rule, termed “early predicted macrolide resistance,” using the evolution of clarithromycin MIC to predict inducible macrolide resistance. Isolates were predicted to be resistant if they had ≥4-fold increase in MIC between day 3 and day 7, with the D7 MIC ≥2 mg/L. Genotypic prediction for macrolide resistance was defined as isolates with a non-truncated *erm*(41), belonging to *M. abscessus* sequevar T28, *M. bolletii,* or possessing mutations in *rrl* A2058/A2059. Isolates identified as *M. massiliense,* presence of truncated *erm*(41), and *M. abscessus* C28 were regarded as genotypically susceptible. We compared “early predicted macrolide resistance”, genotypic resistance, to the day 14 phenotype. Very major error categorizes isolates that were predicted to be susceptible by “early predicted macrolide resistance” method or by genotype, but were, nevertheless, phenotypically resistant on day 14, and major error categorizes isolates that were predicted resistant by proposed rule or genotype but had clarithromycin MIC ≤4 mg/L on day 14.

### Clinical data

We retrospectively extracted the clinical information manually from electronic medical records. The results of DST were retrospectively conducted and not available to the primary care team. We defined patients with evaluable outcomes as those who completed the intended duration of treatment and presented to clinic at 3 months after completion for follow-up. We defined favorable outcomes as sustained clinical cure after the intended antibiotic course without relapse. We defined unfavorable outcomes as a composite outcome for those who died before documentation of cure, or patients who relapsed after treatment completion. Patients with otic infections were cared for by otolaryngologists and diagnosed with chronic otitis media with or without mastoiditis. Patients with bone and sternal infections were grouped together under osteomyelitis; patients with cellulitis, fasciitis, or post-operative wound infections were grouped under SSTI.

### Statistical analysis

Data were expressed as the median with interquartile range for continuous variables, and number and percentage for categorical variables. Statistical analysis was performed using STATA, version 18 (Stata Corporation, College Station, TX, USA). Continuous and categorical variables were compared using the Mann-Whitney *U* and Fisher’s exact tests, respectively. All calculations were two-tailed, and a *P* value of <0.05 was considered statistically significant.

## SUPPLEMENTAL MATERIAL

Table S1 spectrum.00942-25-s0001.docx Comparison of demographics and clinical characteristics on favorable and unfavorable outcome of treatment.

ASM does not own the copyrights to Supplemental Material that may be linked to, or accessed through, an article. The authors have granted ASM a non-exclusive, world-wide license to publish the Supplemental Material files. Please contact the corresponding author directly for reuse.
