# Supplementary material for: Optimizing macrolide resistance detection for Mycobacterium abscessus: a potential low-cost, time-saving alternative
Source: Microbiol Spectr. 2025 Sep 8;13(10):e00942-25. doi: 10.1128/spectrum.00942-25 (PMC12502635; doi:10.1128/spectrum.00942-25)
Supplement: Table S1 — Comparison of demographics and clinical characteristics on favorable and unfavorable outcome of treatment. [file spectrum.00942-25-s0001.docx]

**Table S1. Comparison of demographics and clinical characteristics on favorable and unfavorable outcome of treatment**

| variables | Favorable outcome  N=14 | Unfavorable outcome  N=4 | P |
| --- | --- | --- | --- |
| Demographic characteristics |  | |  |
| Age over 50 y/o | 4 (28.6) | 3 (75.0) | 0.25 |
| Sex (being male) | 6 (42.9) | 3 (75.0) | 0.58 |
| Neutralizing anti-interferon γ autoAbs | 0 | 2 (50.0) | **0.04** |
| MABC subspecies |  | | 0.45 |
| Subspecies *abscessus* | 5 (35.7) | 3 (75.0) |  |
| Subspecies *massiliense* | 8 (57.1) | 1 (25.0) |  |
| SubsutoAbspecies *bolletei* | 1 (7.1) | 0 |  |
| Susceptibility |  |  |  |
| Susceptible to clarithromycin at day 14 | 8 (57.1) | 3 (75.0) | 1.0 |
| Susceptible to amikacin | 13 (92.9) | 4 (100.0) | 1.0 |
| Infection sites |  | | 0.80 |
| Lymphadenitis | 1 (7.1) | 1 (25.0) |  |
| Otic | 5 (35.7) | 1 (25.0) |  |
| Skin and soft tissue infection | 1 (7.1) | 1 (25.0) |  |
| Bone | 3 (21.4) | 1 (25.0) |  |
| Ocular | 2 (14.3) |  |  |
| CNS | 1 (7.1) |  |  |
| Others | 1 (7.1) |  |  |
| Implant or prosthesis involved | 5 (35.7) | 1 (25.0) | 1.0 |
| Treatment entailment |  | | 0.20 |
| <2 effective treatment | 7 (50.0) | 0 |  |
| 2 or more effective treatment | 6 (42.9) | 4 (100.0) |  |
| Topical treatment only | 1 (7.1) | 0 |  |
| Use of macrolides | 13 (92.9) | 4 (100.0) | 1.0 |
| Surgery/debridement involved | 12 (85.7) | 2 (50.0) | 0.20 |
| Treatment duration | 6 (4, 10) | 77.5 (16.5, 131.5) | 0.07 |
| Early prediction of macrolide resistance (predicted susceptible) | 4 (28.6) | 3 (75.0) | 0.25 |
| Genotypic resistance  (predicted susceptible) | 9 (64.3) | 4 (100.0) | 0.28 |
